# The Ace of Spades: Apical Hypertrophic Cardiomyopathy in an African Male Patient

**DOI:** 10.7759/cureus.22514

**Published:** 2022-02-23

**Authors:** Yashvir Rugbeer, Muhammad Ismail, Sandhiran Nadar, Darrin R Naidoo

**Affiliations:** 1 Department of Internal Medicine, Grey's Hospital, Pietermaritzburg, ZAF; 2 Department of Cardiology, Inkosi Albert Luthuli Central Hospital, Durban, ZAF

**Keywords:** magnetic resonance imaging, electrocardiogram, echocardiography, left ventricular hypertrophy, apical hypertrophic cardiomyopathy

## Abstract

We present a case of a 52-year-old African male patient diagnosed with apical hypertrophic cardiomyopathy. He was initially diagnosed with hypertensive heart disease and placed on anti-failure treatment. Following multiple subsequent presentations and on closer review of his signs and symptoms, apical hypertrophic cardiomyopathy was considered. The diagnosis was made five years after his initial presentation and confirmed by echocardiography and cardiac magnetic resonance imaging. This case report explores his presentation and aims to provide further literature on the aetiology, description, and management of apical hypertrophic cardiomyopathy, particularly within the African population.

## Introduction

Hypertrophic cardiomyopathy (HCM) describes myocardial disease with left ventricular hypertrophy (LVH) in the absence of a precipitating cardiac or systemic cause [1]. HCM is generally well described, and although likely to be evenly distributed among different populations worldwide, it is noted to be especially rare in the African setting [1,2]. Further literature of its incidence, prevalence and presentation within the African population is required, which will ultimately guide effective management and prevention strategies [2]. Apical hypertrophic cardiomyopathy (Ap HCM) is a rare variant of HCM, and was first described in a case report published in Japan in 1976 [1,3,4,5]. In the largest study of a cohort of patients with HCM who had undergone investigation, a <10% prevalence of Ap HCM was noted [1,6,7,8]. The mean age of presentation of Ap HCM is 41 ± 14.5 years, and has a male to female ratio of 1:5 [9].

In a retrospective study of 105 patients diagnosed with Ap HCM conducted in Toronto in 2002, 56% of patients were symptomatic upon diagnosis and follow-up [10]. The commonest symptom experienced was exertional dyspnoea (38%), followed by palpitations (21%), typical angina (18%), atypical chest pain (14%), pre-syncopal episodes (10%) and syncopal episodes (6%) [10]. Examination findings include a sustained, forceful double apical impulse, which is laterally displaced [11]. Auscultation may reveal a normal first heart sound (S1), a split second heart sound (S2), and an audible fourth heart sound (S4), with or without a crescendo-decrescendo, mid-diastolic murmur heard loudest at the lower left sternal border [4,9,11]. Decompensated left ventricular function and subsequent pulmonary hypertension can present with the classic clinical signs and symptoms of bi-ventricular failure, such as exertional dyspnoea, orthopnoea, paroxysmal nocturnal dyspnoea, a raised jugular venous pulsation, and the presence of a third heart sound (S3) [12]. The complications of Ap HCM, as noted in the aforementioned retrospective Toronto study in 2002, included atrial fibrillation (12%), myocardial infarction (10.5%), congestive cardiac failure (5%), and ventricular fibrillation (1%) [10]. Overall mortality was noted to be 10.5%, with a cardiovascular mortality of 1.9% and an annual cardiovascular mortality of 0.1% [10]. Diagnostic criteria include asymmetrical left ventricular hypertrophy mostly confined to the apex of the heart, an apical wall thickness of ≥ 15mm, and a ratio of maximal apical to posterior wall thickness of ≥ 1.5mm based on echocardiography (ECHO) or cardiac magnetic resonance imaging (MRI) [1,4,6,9]. Typical electrocardiography (ECG) findings are negative T-waves in the precordial leads with a depth of ≥ 10mm, and evidence of left ventricular hypertrophy [3,9]. Cardiac ventriculography may demonstrate the typical “ace-of-spades”-like configuration in the left ventricle, along with marked apical obliteration [1]

## Case presentation

A 52-year-old African male patient with no known medical co-morbidities or prior cardiac history presented to the emergency department of a tertiary level hospital in September 2016, having been referred from his base hospital for further investigation and management. His symptoms included a one-day history of progressively worsening typical cardiac chest pain (retrosternal chest discomfort worsened by exertion and relieved by rest [13] ), nausea, and diaphoresis. Table 1 summarizes his relevant clinical findings on the initial presentation. 

**Table 1:** Clinical findings on presentation

Category	Clinical Findings
Vital signs	Pulse: 60 beats per minute, regular rhythm and normal volume, blood pressure: 146/92 mmHg, respiratory rate: 18 breaths per minute, temperature: 36.8 ⁰C, oxygen saturation: 98% in ambient room air, blood glucose: 5.8 mmol/L
General examination	Comfortable at rest. Absence of clubbing, pallor, and pedal oedema. No signs of central or peripheral cyanosis, plethora or diaphoresis.
Cardiac system examination	No signs of infective endocarditis. A non-elevated, normal morphology jugular venous pulsation. Normal precordium, no parasternal heave, thrills, or palpable pulmonary component of the second heart sound (P2). Displaced apex beat to the left 6^th^ intercostal space in the anterior axillary line. Normal first heart sound (S1) with a loud aortic component of the second heart sound (A2). No additional heart sounds/murmurs.
Respiratory system examination	Chest fields clear with good air entry bilaterally. No crepitations or features of costochondritis.
Abdominal system examination	Soft and non-tender. No organomegaly.
Nervous system examination	Oriented to time, person and place. No focal neurological signs.

**Figure 1:**
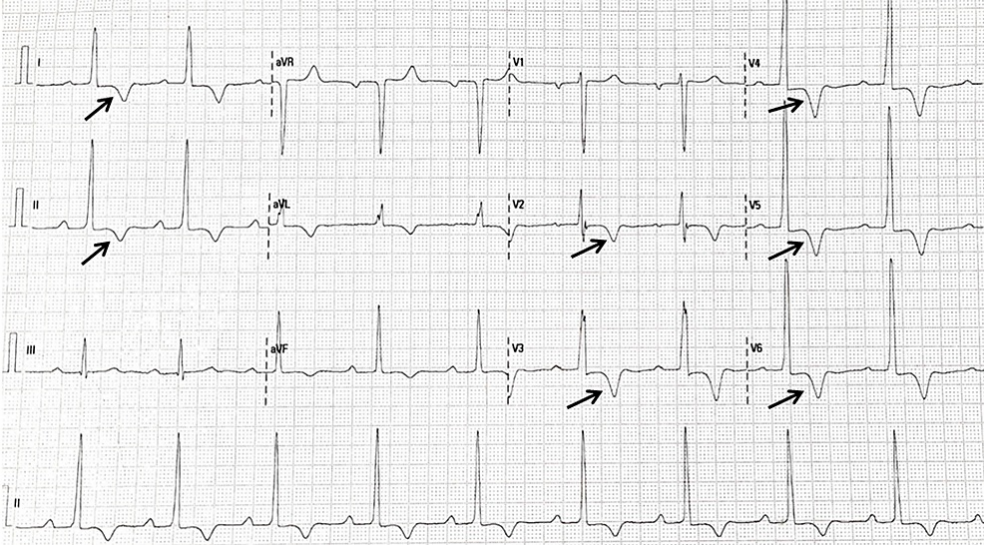
ECG of the patient on initial presentation A sinus rhythm with a rate of 60 beats per minute and a normal electrical axis is noted, along with left ventricular hypertrophy according to voltage criteria and 1mm ST-segment depression. Arrows illustrate inverted T-waves of > 5mm in Leads I, II, and V2-V6.

**Figure 2:**
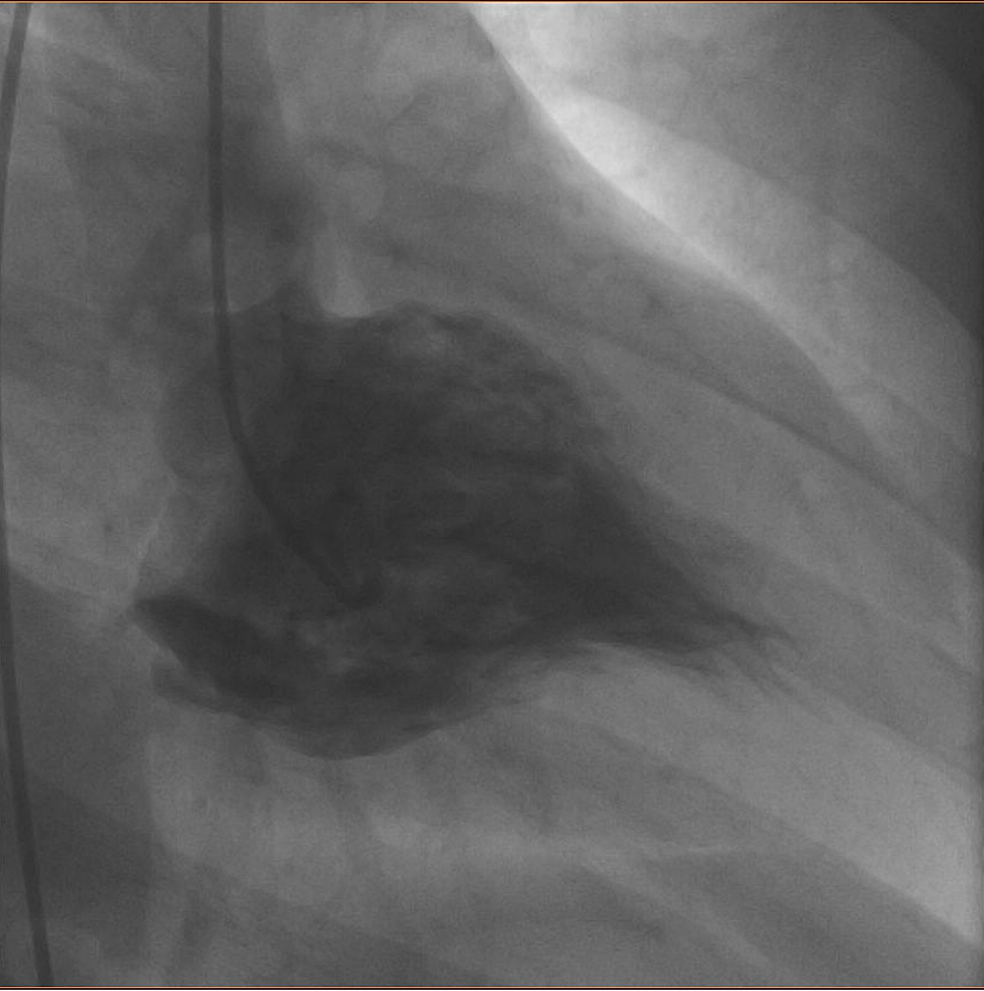
Left ventriculography performed on the patient The image illustrates a typical "ace-of-spades"-like configuration of the left ventricle.

Laboratory investigations were normal, apart from an elevated cardiac troponin-I level of 1185 ng/L taken 16 hours following the onset of chest pain, and an elevated creatine kinase (CK) level of 268 U/L. The creatine kinase MB (CK-MB) mass was of a normal value of 2.70 ug/L, and a repeat troponin-I level taken six hours later was of a value of 1122 ng/L (a 5% decrement from the initial value). Figure 1 illustrates the electrocardiogram (ECG) taken on initial presentation and describes its findings. Echocardiography noted an end-diastolic left ventricular internal dimension measured at 4.9 cm (normal range: 4.2 cm - 5.8 cm), an ejection fraction of 65%, and no valvular or pericardial abnormalities. Cardiac angiography noted normal epicardial arteries with a hocoid left ventricle; left ventriculography showed a typical "ace-of-spades"-like configuration of the left ventricular cavity (See Figure 2).

**Figure 3:**
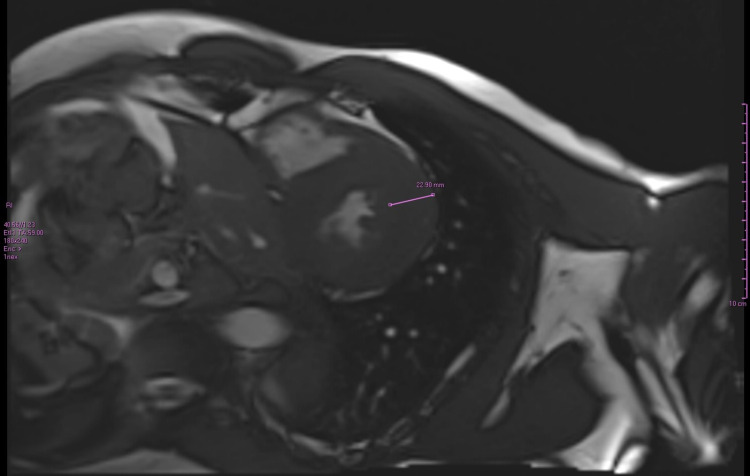
Cardiac MRI of the patient Cine image of the short oblique-axis of the left ventricle. The thickness of the basal anterior lateral wall (purple arrow span) is measured at 23mm (normal values of 6.5mm - 8.5mm), demonstrating significant myocardial thickening.

**Figure 4:**
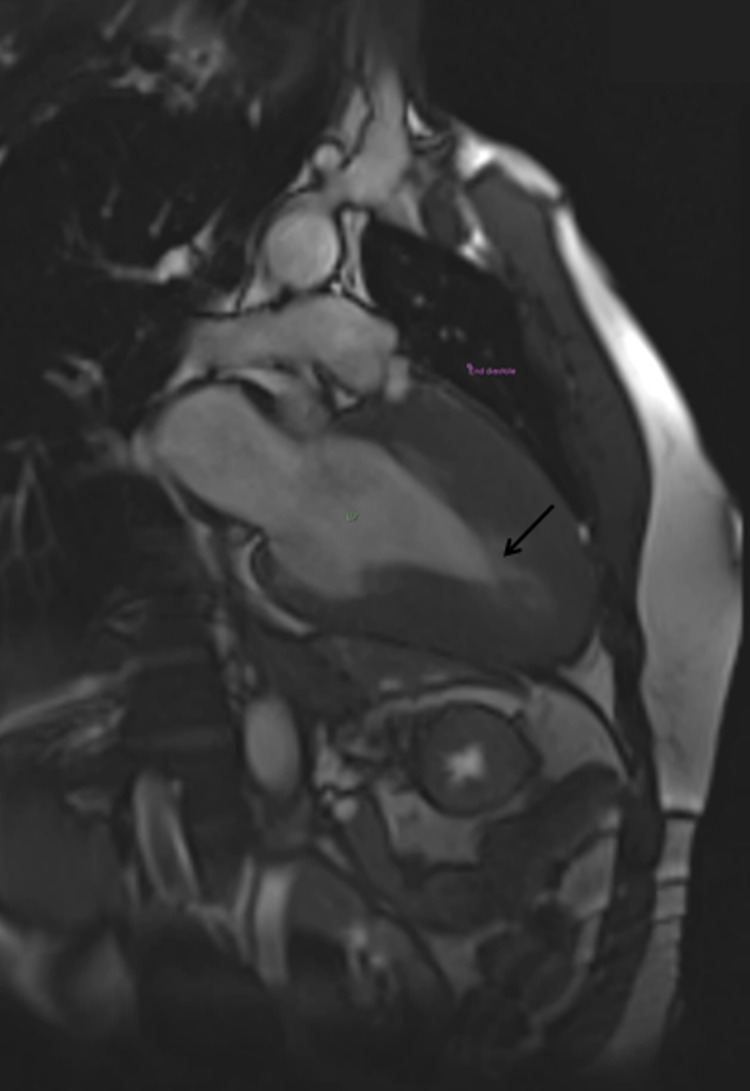
Two-chamber view (end-diastole) Arrow indicates near-complete cavity obliteration of the apex in end-diastole.

**Figure 5:**
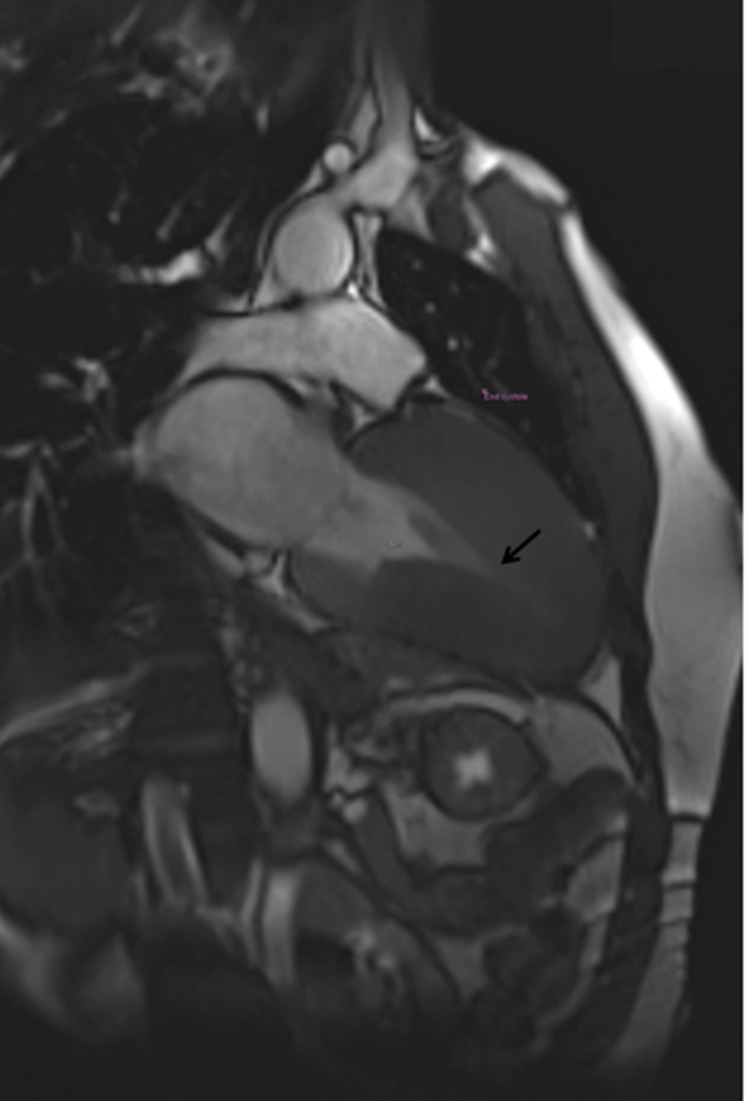
Two-chamber view (end-systole) Arrow indicates near-complete cavity obliteration of the apex in end-systole.

**Figure 6:**
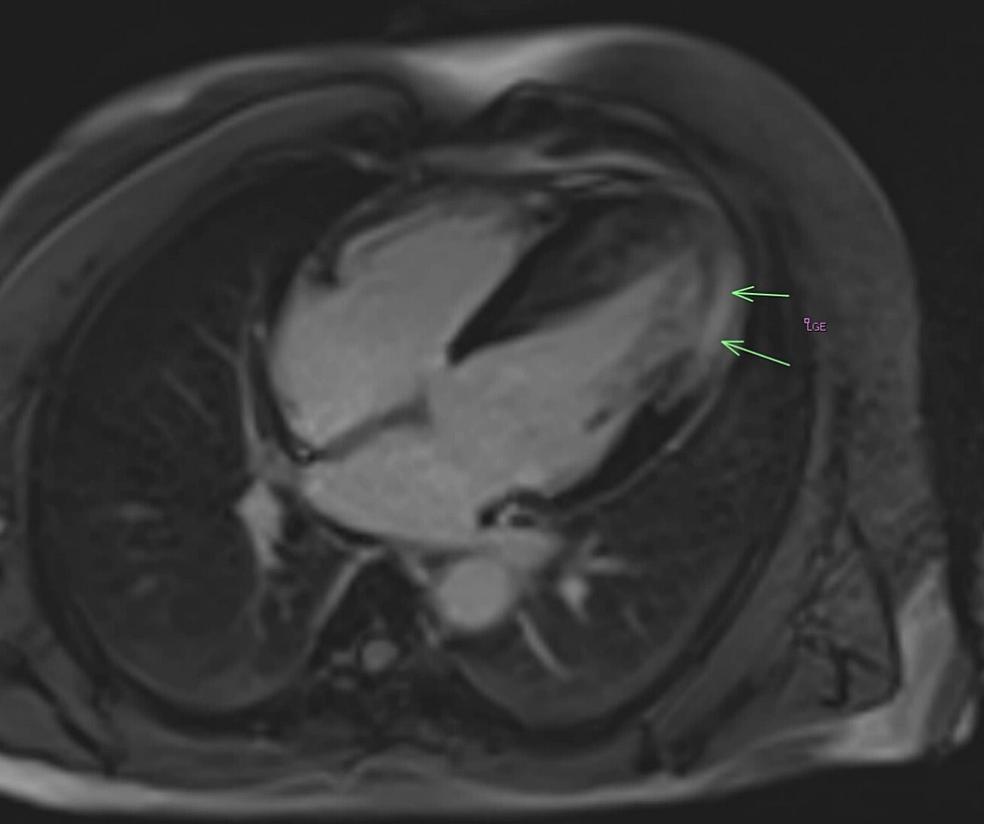
Four-chamber view with gadolinium enhancement Arrows indicate late gadolinium enhancement: Significant mid-wall myocardial enhancement of the mid-ventricle and apical lateral wall is observed, indicating significant fibrosis.

An initial diagnosis of hypertensive heart disease with elevated serum troponin levels was made. The patient was discharged and placed on enalapril 2.5mg bi-daily, aspirin 150mg daily, and atorvastatin 40mg daily. He presented a further two times with similar presentations but was again discharged with the above medication. In September 2020, he once again presented to the emergency unit, now complaining of pre-syncopal symptoms and multiple syncopal episodes. On close review of his investigative profile along with repeat ECG and echocardiography, a diagnosis of Ap HCM was considered. Cardiac MRI was performed, which noted significant myocardial wall thickening of 23mm (See Figure 3), and near-complete cavity obliteration of the apex (See Figure 4 and Figure 5). Features were in keeping with predominant apical hypertrophic obstructive cardiomyopathy, along with myocardial infiltration and fibrosis (See Figure 6). A definitive diagnosis of Ap HCM was reached, and the patient was placed on Atenolol 25mg daily and Amiodarone 400mg daily. He unfortunately demised six months later, with sudden cardiac death being the presumed cause. 

## Discussion

Multiple different morphologically distinct patterns of hypertrophic cardiomyopathy are described [1]. The commonest sub-types are asymmetrical septa, concentric, reverse septal, neutral, and apical, along with other rarer forms [1]. It is further sub-divided into three types, being either pure focal, pure diffuse, or a combination of the two [1]. The aetiology of Ap HCM is hereditary, with an autosomal dominant mode of inheritance [7,14]. The condition results from mutations in sarcomeric protein genes; however, these mutations were found in less than a quarter of patients diagnosed with the disease who had undergone genetic studies [7]. The commonest genotypes were noted to be mutations in the myosin-binding protein C (MYBPC3) gene, and the myosin heavy-chain beta 7 (*MYH7*) gene located on chromosome 11q11 [7,14]. The diagnostic approach involves a comprehensive assessment of the patient, including close attention to symptoms and clinical examination. Investigations such as ECG, ECHO, and MRI may confirm the diagnosis, with risk stratification via the American College of Cardiology/European Society of Cardiology (ACC/ESC) algorithm also necessary [1,9]. Management options are categorized into optimal medical treatment, surgery, and ablation, as well as specialized cardiac devices such as implantable cardioverter-defibrillators [9]. Medications such as beta-blockers, calcium channel blockers, and antiarrhythmic agents are commonly used in symptomatic patients, with the objectives of alleviating symptoms as well as decreasing the incidence of events such as arrhythmias and sudden cardiac death [9]. Angiotensin-converting-enzyme (ACE) inhibitors, angiotensin receptor blockers (ARBs), and aldosterone receptor antagonists are not recommended, as their action may exacerbate symptoms and further predispose to pre-syncopal/syncopal episodes by further reducing afterload and increasing outflow gradient [15]

The diagnosis of Ap HCM was only considered five years following our patient's initial presentation. This could be due to the relative rarity of the disease in the African population combined with the challenge of severely resource-limited settings. The hospital where the patient was eventually diagnosed and managed provides a tertiary level healthcare service (i.e., specialised health care) to approximately 3.5 million patients across five different health districts. This results in extremely limited access to investigative resources (such as ECHO and MRI), as well as specialist cardiology consultation. Unfortunately, the delay in diagnosis along with constrained management options subsequently contributed to the poor outcome seen in our patient. The lack of availability of genetic testing for the condition in our setting also results in difficulty in excluding the disease in first degree relatives, where early diagnosis would prevent long-term morbidity and mortality. 

## Conclusions

Ap HCM introduces an interesting caveat to the spectrum of HCM, and is known to be of very low incidence in the African population. Diagnosis requires a high index of suspicion and close attention to a patient's profile, with early surveillance and management necessary to reduce long-term morbidity and mortality. Due to its genetic basis, this not only holds true for the affected patient, but also for first degree relatives. We suggest that Ap HCM be more frequently considered with suggestive patient presentation and examination, along within the disease spectrum of HCM. Further research and literature of the condition, especially within the African population, would prove to be of value. 
